# Superior adsorption and photoinduced carries transfer behaviors of dandelion-shaped Bi_2_S_3_@MoS_2_: experiments and theory

**DOI:** 10.1038/srep42484

**Published:** 2017-02-13

**Authors:** Mengjiao Li, Junyong Wang, Peng Zhang, Qinglin Deng, Jinzhong Zhang, Kai Jiang, Zhigao Hu, Junhao Chu

**Affiliations:** 1 Technical Center for Multifunctional Magneto-Optical Spectroscopy (ECNU), Shanghai Department of Electronic Engineering, East China Normal University, Shanghai 200241, China

## Abstract

The enhanced light-harvesting capacity and effective separation of photogenerated carriers in fantastic hierarchical heterostructures enjoy striking attention for potential applications in the field of solar cells and photocatalysis. A three-dimensional (3D) dandelion-shaped hierarchical Bi_2_S_3_ microsphere compactly decorated with wing-shaped few layered MoS_2_ lamella (D-BM) was fabricated via a facile hydrothermal self-assembly process. Especially, polyethylene glycol (PEG) has been proven as the vital template to form D-BM microsphere. Importantly, the as-prepared D-BM microsphere presents pH-dependent superior adsorption behavior and remarkable visible light photocatalytic activity for degradation of organic dyestuffs (Rhodamine B/RhB and Methylene blue/MB), far exceeding those for the pure Bi_2_S_3_ and MoS_2_. It is understandable that D-BM with high surface area possesses more active sites and promotes light utilization due to the unique porous structure with outspread wings. Besides, based on the experiments and theoretical calculations, the staggered type II band alignment of D-BM permits the charge injection from Bi_2_S_3_ to MoS_2_, subsequently accelerates the separation and restrains the recombination of carriers, leading to excellent photocatalytic activity, as well as the photoconductance and photoresponse performance (with *I*_light_/*I*_dark_ ratio 567).

The intense demand for photocatalysis applying to the pollutants degradation and the effective solar energy conversion keeps moving due to the worsening environment and energy crisis^1^,^2^,^3^,^4^,^5^,^6^,^7^,^8^,^9^. It is believed that the desired photocatalysis should have high light-harvesting capability and retardative recombination of photoexcited carriers^10^,^11^,^12^. A promising approach to enhance the photoactivity is to increase the light absorption range, the surface area and active site of the catalysts. Numerous studies have shed light on the importance of the semiconductor-based composites with certain modification in improving the performance, such as photovoltaic conversion, catalytic, and electrochemical properties^13^,^14^,^15^,^16^. Thus, studying the hierarchical semiconductor nanocomposite with controllable morphologies becomes the frontier strategy. Among these semiconductors, mater sulfides (ZnS, CdS) have been extensively surveyed for the generation of new optoelectronic performance with effective electron-hole separation and transport^5^,^14^.

Typically, as a eco-friendly lamellar-structured semiconductor, Bi_2_S_3_, with a typical band gap (1.3–1.7 eV) for solar photovoltaic, has attracted lots of research interests^17^,^18^,^19^. In recent years, reported Bi_2_S_3_ nanocrystal with various morphologies have promising applications in solar cells, photodetectors, gas sensors, electrochemical hydrogen storage and X-ray computed tomography imaging due to the suitable band gap^20^,^21^,^22^,^23^. Besides, it also has been used as a stability sensitizer and photocatalyst derived from the broad absorption of visible light up to 800 nm^24^,^25^. However, the photocatalytic performance of Bi_2_S_3_ has been restricted because of the toilless recombination of photogenerated carriers and therewith the low quantum yield. Hence, as a feasible method so far to prolong the lifespan of electron-hole pairs, establishing composite structures with metal oxides, metal chalcogenides or 2-dimensional (2D) hexagonal graphene, for instance Bi_2_S_3_/Bi_2_WO_6_, Bi_2_S_3_/TiO_2_ and Bi_2_S_3_/Pd_4_S, has been paid significant attention^12^,^26^,^27^,^28^,^29^. In particular, the 3-dimensional (3D) porous architecture with Bi_2_S_3_ skeleton, keeping superiority in large active contact area, is considered to have potential in synergistically enhancing the photocatalytic features.

The wide researches and successful applications of graphene recently have triggered great attention on 2D-layered materials. The 2D transition metal dichalcogenides (TMD) exhibit ultrathin physical characteristic, excellent optical, electronic, mechanical properties and potential applications in field-effect transitions (FET) and sensing devices^30^,^31^,^32^,^33^,^34^. As a representative 2D TMD, consisted of the weakly coupled S-Mo-S atoms sandwich layers, MoS_2_ nanostructures have been synthesized with plentiful morphologies of nanosheet, nanoribbon and nanosphere^35^,^36^,^37^,^38^. Different from graphene, the controlled band gap (1.2–1.9 eV) and thermal stability, make MoS_2_ be widely applied to biosensors, memory, capacitors, logic circuit devices, and lithium batteries^39^,^40^,^41^. Importantly, 2D-layered MoS_2_ possesses a large surface area and massive active sites, which can provide sufficient contact and effective reactions^42^. Hence, forming hierarchical composites with MoS_2_ has been proven to be promising and it also opens opportunities for optoelectronic applications. Zhou *et al*. have successfully fabricated MoS_2_ nanosheet-coated TiO_2_ nanobelt heterostructures, which showed a high photocatalytic hydrogen production and strong photocatalytic degradation of the dye molecules^31^. It is believed that the formation of this MoS_2_-TiO_2_ nanocomposite forcefully retard the photogenerated electron-hole recombination. Moreover, Chen *et al*. have put forward a bottom-up strategy of solvothermal method for 2D MoS_2_ nanosheets composites, which exhibited preeminent properties in biomedicine and realized considerable applications in photothermal regression of tumor^43^,^44^.

In this work, we propose a novel heterostructure of 3D-dandelion Bi_2_S_3_@MoS_2_ microsphere using a facile hydrothermal method for the first time. Intriguingly, the layers of MoS_2_ coating likes the wring of the dandelion. This unique architecture offered a high light utilization as a result of large surface area and massive active sites for reactions. Moreover, the formation of the staggered type II band alignment in D-BM made a prolonged lifespan of electron-hole pairs, based on calculated energy band positions and the corresponding electronic structures. Obviously, evaluated by the degradation of Rhodamine B (RhB) and Methylene blue (MB) under visible light, the as synthesized heterostructure exhibits more remarkable adsorption and visible light photocatalytic properties than the pristine Bi_2_S_3_ and MoS_2_. Also, it presents improved photoresponse property with a high *I*_light_/*I*_dark_ ratio of 567.

## Results and Discussion

### Phase structure and Raman studies

**Figure 1:**
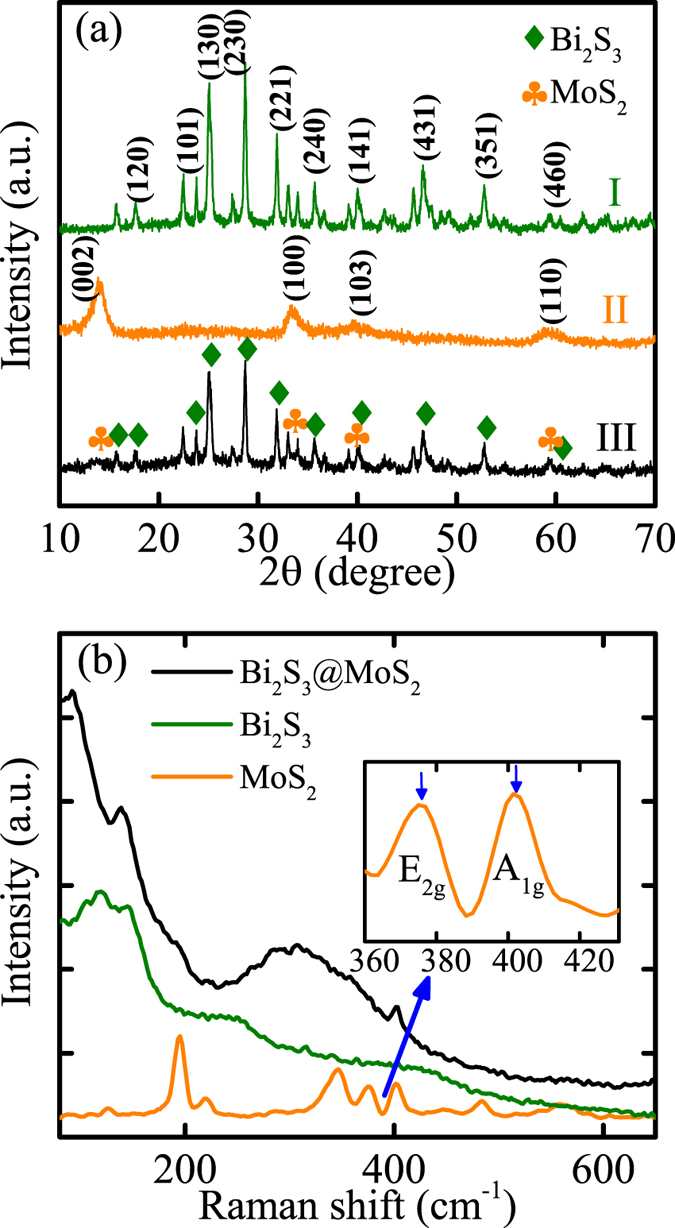
(**a**) XRD patterns and (**b**) Raman spectra of pristine of Bi_2_S_3_, MoS_2_, and the D-BM composites. The inset shows both E_2*g*_ and A_1*g*_ Raman modes of MoS_2_.

As shown in Fig. 1a, the phase information and purity of D-Bi_2_S_3_, MoS_2_ nanoflowers and their composites were characterized by XRD analysis. Pattern I corresponded well to the lattice planes of orthorhombic Bi_2_S_3_ (JCPDS#65-2431 *a* = 11.290 Å, *b* = 3.978 Å, and *c* = 11.150 Å) without some impurity as Bi_2_O_3_. And pattern II can be indexed to the standard peaks of hexagonal MoS_2_ (JCPDS#37-1492; *a* = 3.16 Å, *b* = 3.16 Å, and *c* = 12.30 Å). As for hierarchical D-BM structure (5 MBS, pattern III), the weak diffraction peak at 2θ = 14.2°, corresponding to the MoS_2_ lattice plane of (002), can be inferred that the few-layered MoS_2_ petals have been broken by D-Bi_2_S_3_. Raman spectroscopy was further utilized to identify the hybrid production. In Fig. 1b, two characteristic peaks of MoS_2_ can be observed at 375 and 402 cm^−1^ from the local amplification, which correspond to the E_2g_ and A_1g_ vibration modes, respectively^41^. The vibrational modes of pure Bi_2_S_3_ microspheres are located at 107 cm^−1^ and 260 cm^−1^, which can be assigned as the A_g_ and B_1g_ modes^21^. Obviously, all the characteristic Raman spectral signatures of Bi_2_S_3_ and MoS_2_ both are presented in the result of their hybrids, demonstrating the successful incorporation of D-BM structures. Note that there is a blue shift of the B_1g_ mode compared to the pure Bi_2_S_3_. It probably suggests the surface strain is due to the novel coated MoS_2_ on each Bi_2_S_3_ nanorod.

### Morphology analysis

**Figure 2:**
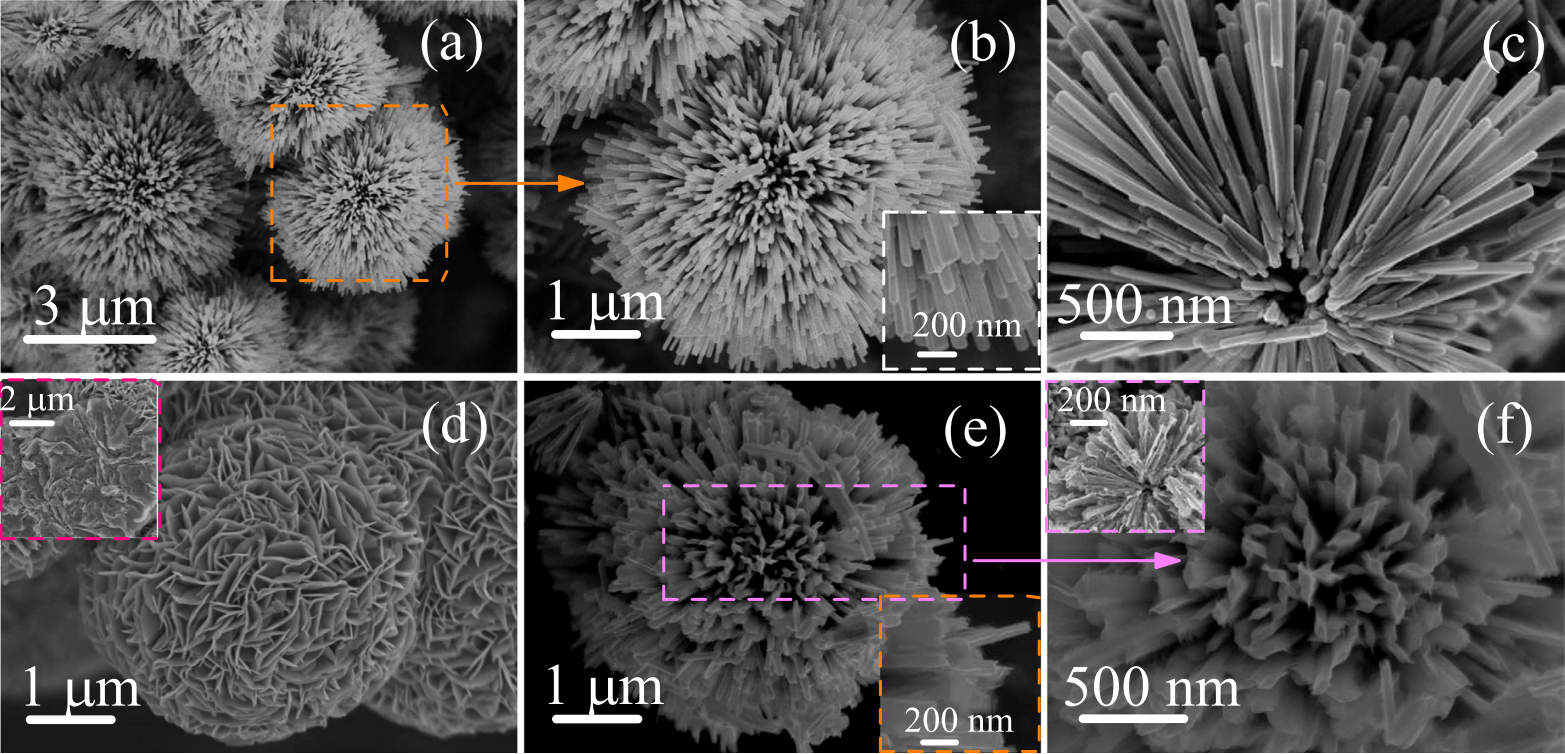
(**a**,**b**) SEM images of the dandelion-shaped Bi_2_S_3_ microspheres under different magnifications. The inset shows the high magnification SEM image of vimineous Bi_2_S_3_ nanorods. (**c**) The broken Bi_2_S_3_ microspheres. (**d**) SEM image of MoS_2_ nanoflower and the inset is the several chapped MoS_2_ nanoflowers. (**e**,**f**) SEM image with different magnifications of the synthesized D-BM heterostructures. The insets are a local amplification from the side views and a broken section.

The morphology and micro-structure of all the samples have been investigated by SEM images. Figure 2a,b display the pure D-Bi_2_S_3_ microspheres in different magnifications. It can be seen that the Bi_2_S_3_ structures are distributed in the shape of irregular microspheres with the average diameter of 5 *μ*m. Apparently (inset of Fig. 2b), these D-Bi_2_S_3_ micropheres were composed of a large number of acicular crystalline nanorods with uniform diameters of about 80 nm. Moreover, the broken D-Bi_2_S_3_ microsphere shown in Fig. 2c indicates that the acicular Bi_2_S_3_ nanorods radiate from the center and stack uniformly. It is extremely vital that the interstices between Bi_2_S_3_ nanorods can provide particular framework for the embedded of layered MoS_2_ petals. As presented in Fig. 2d, the pure MoS_2_ flowers reveal the diameter of 4 *μ*m, with number of thinnish petals aggregated closely. The inset of Fig. 2d provides a chapped MoS_2_ flowers, and it illustrates that these disordered petals grown from a common center to form the spherical structure. Figure 2e exhibits the morphology of the hydrothermal synthesized D-BM (5 MBS) hetero-microspheres. Detailedly, some other SEM information on Bi_2_S_3_, MoS_2_, and hybrids are shown in Figs S1 and S2 (ESI). Generally, the whole D-Bi_2_S_3_ microspheres are uniformly covered with 2D MoS_2_ nanosheets. A high magnification top view SEM image shown in the inset of Fig. 2e, the composites present apparent differences from the pristine D-Bi_2_S_3_ or MoS_2_. Iconically, almost each Bi_2_S_3_ nanorod are compactly decorated with a pair of expanding wings of MoS_2_ nanosheets, thus forming the MoS_2_ coated Bi_2_S_3_ heterostructure. In the inset of Fig. 2f, a section of the broken composites elucidates that MoS_2_ nanosheets have grown along the Bi_2_S_3_ nanorods and deeply rooted in the center of Bi_2_S_3_ spheres, which expects that this unique hierarchical architecture can provide more effective activity sites and enhance the photoelectric properties.

**Figure 3:**
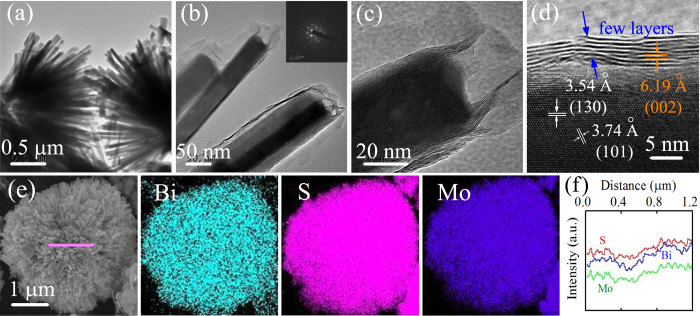
(**a**) TEM image of D-BM microstructures. (**b**) The surface of D-BM and the inset is the SAED pattern. (**c**,**d**) HRTEM images of D-BM. (**e**) SEM image of D-BM and the corresponding EDS mapping images of Bi, S, and Mo elements. (**f**) The corresponding EDS line scan along the pink line in (**e**).

In addition, a sequence of TEM and HRTEM images of D-BM nanocomposite have been employed to reveal more specific structural information. In Fig. 3a, homogeneous MoS_2_ sheets are detected at the edge of each Bi_2_S_3_ nanorod from the low magnification TEM survey. Not only Fig. 3b,c clarify the intimate interfacial contact between MoS_2_ sheets and elongated Bi_2_S_3_ rods, but also they indicate that the MoS_2_ sheets are ultrathin compared with the pure MoS_2_ flowers. In the HRTEM image (Fig. 3e), the lattice fringes of *d* = 3.54 Å and *d* = 3.74 Å, corresponding to the (130) and (101) planes of Bi_2_S_3_, respectively^28^. The coated MoS_2_ exhibits the lattice spacing of 6.19 Å, which matches well with the (002) planes of hexagonal MoS_2_^31^. Furthermore, the related cleaved crystal structure in theoretical section indicates the distance between adjacent Bi atoms on the (130) crystal surface of Bi_2_S_3_ (3.4148 nm), which is eleven intervals of the adjacent S atoms on the (001) crystal surface of MoS_2_ (0.3169 nm × 11)^45^. It is believed that Bi_2_S_3_ nanorods might be available for the growth of the MoS_2_ nanosheets to form the heterostructure between the mutual effect of S and Bi atoms. Thus, it can be inferred that the MoS_2_ sheets, with about 5–8 layers, embellished at the surface of the Bi_2_S_3_ rods. Moreover, the selected area electron diffraction (SAED) pattern (inset of Fig. 3b) further proves the mixed-phase nature of single crystal Bi_2_S_3_ (bright diffraction spots) and layered superimposed MoS_2_ sheets (diffraction rings). In order to accurately confirm the elemental composition and spatial distribution, energy dispersive X-ray spectrometry (EDS) analysis in Fig. 3e have been performed. The well-proportioned distributions of S, Bi, and Mo can be obtained from the mapping results. Besides, the EDS line scan (Fig. 3f) of the marked region sheds light on the unique hierarchical heterostructure, as well as in agreement with SEM and TEM observation.

### XPS analysis

**Figure 4:**
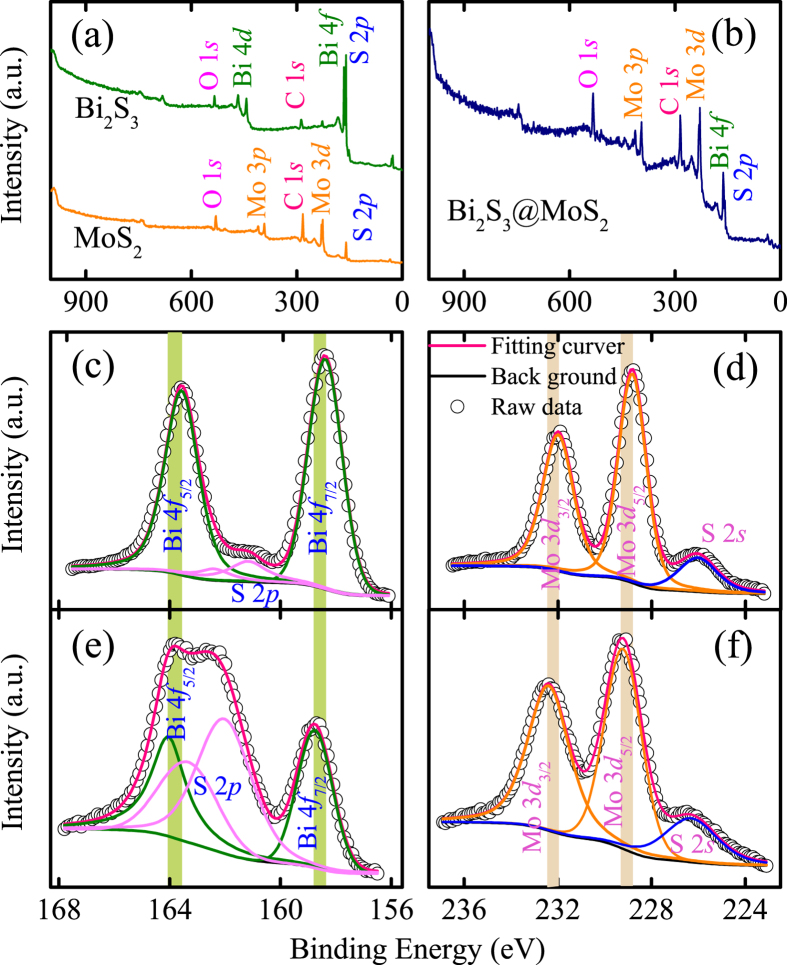
XPS spectra of as-synthesized products. (**a**,**b**) The overall spectra of Bi_2_S_3_, MoS_2_, and 5 MBS, respectively. The Bi 4*f* and Mo 3*d* spectra for Bi_2_S_3_ (**c**), MoS_2_ (**d**), and D-BM (**e**,**f**), respectively. Note that the scatter and solid lines indicate the experimental data and fitting results, respectively. The dotted lines mark the peak shifts of Bi 4*f* and Mo 3*d*.

We also performed XPS analysis to elucidate the surface chemical composition and valence states of the pristine Bi_2_S_3_, MoS_2_, and D-BM (5 MBS) heterostructures. The Fig. 4a shows the survey XPS spectra of Bi_2_S_3_ and MoS_2_ (Note that C element acts as reference and O element comes from the absorbed oxygen). After the second hydrothermal reaction, the overall XPS spectra in Fig. 4b indicates the main constituent of Bi, S, and Mo elements. Figure 4c,d display the spin-orbit components of Bi 4*f* (158.46/163.76 eV) and Mo 3*d* (228.84/231.99 eV) for pure Bi_2_S_3_ and MoS_2_, respectively. In addition, the XPS spectrum of the hybrids in Fig. 4e can be well fitted into several dominate peaks with binding energies of 158.75 eV, 162.05 eV, 163.30 eV, and 164.05 eV, which are assigned to Bi 4*f*_7/2_, S 2*p*_3/2_, S 2*p*_1/2_, and Bi 4*f*_5/2_, respectively. The high resolution Mo 3*d* (Fig. 4f) binding energies of the hybrids are located at around 229.23 eV and 232.38 eV, corresponding to Mo 3*d*_5/2_ and Mo 3*d*_3/2_, respectively. Notablely, the Bi 4*f* and Mo 3*d* peaks shift toward the high banding energy, with dotted lines mark. Hence, it could be deduced that chemical bonds of Bi-S-Mo formed probably, related to the electronic shielding effect^46^. A weak peak at 226.31 eV has also be found (Fig. 4f), which is attributed to the S 2*s*^47^. Take the spin orbit separation into consideration, the phenomenon among Bi 4*f* (5.30 eV), S 2*p* (1.25 eV), and Mo 3*d* (3.15 eV) peaks disclose the existence of Bi^3+^, S^2−^, and Mo^4+^, based on the reported results.

### Growth mechanism investigation

**Figure 5:**
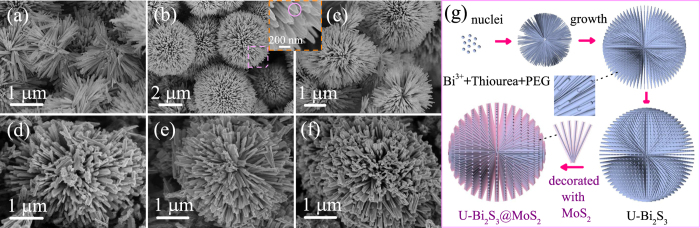
(**a**–**c**) SEM images of the Bi_2_S_3_ products fabricated at 1 h, 3 h, and 8 h, respectively. (**d**–**f**) SEM images of the D-BM products obtained at 5 h, 8 h, and 12 h, respectively. (**g**) Schematic illustration of the morphological evolution process of D-BM hybrids.

The growth mechanism of the D-BM nanomaterials has been studied for potential applications and controllable synthesis of other novel structures. For D-Bi_2_S_3_, the formation process has been investigated by SEM at different reaction times, as shown in Fig. 5a–f. At the early reaction stage (1 h), the half-baked micropheres (diameter of 2 *μ*m) have been formed and proven to be orthorhombic Bi_2_S_3_ by XRD analysis (Fig. S3), which indicate the fast nucleation of Bi_2_S_3_. As the reaction proceeded (3 h), some actinomorphic-shaped Bi_2_S_3_ (diameter of 5 *μ*m) can be captured in Fig. 5b. From the close inspection (Fig. S4), however, the surface of the radial Bi_2_S_3_ nanorods are rough and inhomogenous, indicating a rapid growth of Bi_2_S_3_ microspheres. By prolonging the reaction time to 8 h, massive intact D-Bi_2_S_3_ microspheres have been successfully synthesized. Then the products under hydrothermal condition have been collected at 5 h, 8 h, and 12 h. The SEM images in Fig. 5d–f manifest that the coated MoS_2_ nanosheets become increasing and orderly along with the prolonged reaction process. It can also be verified through the related XRD survey (Fig. S4). At the beginning, the characteristic peak of MoS_2_ at 2θ = 14.2° is indeed indetectable. With prolonging the reaction time, the emergence of MoS_2_ (002) plane provides the convincible proof for the formation of the MoS_2_ coated Bi_2_S_3_ nanocomposites.

Based on the results of the time-dependent experiments and analysis, the probable morphology evolution process of the hetero-Bi_2_S_3_@MoS_2_ structure is illustrated in Fig. 5g. In our experiments, thiourea was chosen to act as the sulfide source, for constituting the Bi^3+^-thiourea complexes, initially. Under elevated temperature, these complexes decomposed, accompanied by shaping into needle-like nanospheres with nucleated Bi_2_S_3_^48^,^49^. As time went on, Bi_2_S_3_ grew gradually to improve the rough surface, at the expense of Bi_2_S_3_ particles or rods. It can be attributed to the typical Ostwald ripening process, which could significantly reduce the total surface free energy^50^. Besides, the solution of PEG is necessary to build a suitable viscous surroundings, which promotes the unique geometrical patterns. For comparison, the morphology and crystallinity of the product obtained without PEG also have been surveyed. As a result (Fig. S5), the regular Bi_2_S_3_ microspheres with short tomentum ascertain the role of PEG as the important template. Finally, the 3D D-Bi_2_S_3_ microspheres could provide available high active sites for the growth and insertion of MoS_2_ nuclei, leading to the hierarchical and porous framework.

### BET surface areas and photocurrent response

**Figure 6:**
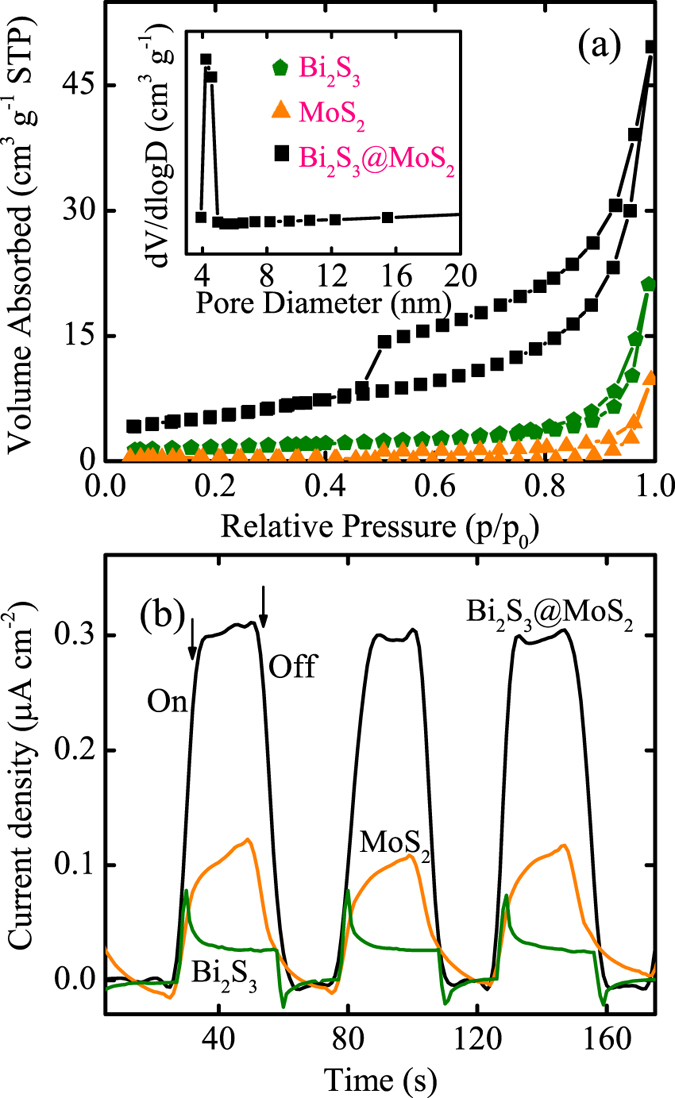
(**a**) Nitrogen adsorption-desorption isotherms and the pore-size distribution (inset) of the as-synthesized samples. (**b**) Comparison of transient photocurrent responses of the as-prepared products illuminated by simulated sunlight.

As shown in Fig. 6a, the nitrogen adsorption-desorption measurements have been performed to ascertain the surface area and the porous structure of the as-synthesized samples. According to the Brunaner-Deming-Deming-Teller (BDDT) classification, the isotherms of D-BM displays a typical hysteresis loops as type IV. The specific Brunauer-Emmett-Teller (BET) surface area have been estimated to be about 19.48 m^2^/g for the hybrid. Obviously, the characteristics with enlarged surface area and porosity of the composite recognized the embedded structure of MoS_2_ nanosheets into Bi_2_S_3_ microspheres. By the Barrett-Joyner-Halenda analysis, the pore-size distribution of Bi_2_S_3_@MoS_2_ (inset of Fig. 6a) indicates the main mesopores with about 4.5 nm.

Before moving toward the photocatalytic study of the as-synthesized products, the separation of charge carriers, as a crucial factor, needs to be investigated indispensably. Hence, the photocurrent transient response measurements of pure Bi_2_S_3_, MoS_2_, and their mesoporous composites have been carried out to verify the extended lifespan of the photogenerated charges. Under visible-light irradiation, Fig. 6b records the fast and consistent photocurrent responses over several on-off cycles, illustrating that all three samples are reproducible and stable. Apparently, pure Bi_2_S_3_ and MoS_2_ both present low photocurrent densities, consisted with the low quantum efficiency. However, the photocurrent density of the D-BM electrode is enlarged about one order of magnitude higher than the pure Bi_2_S_3_ electrode. It can be interpreted that this novel heterostructure possess particular tunnel for transformation of photogenerated carries, subsequently retards the recombination and extends the lifetime of carries.

### Adsorption and photocatalytic properties

**Figure 7:**
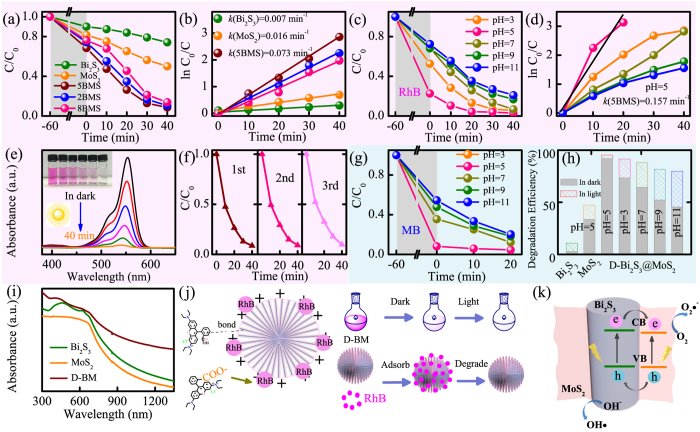
The adsorption (in dark) and photodegradation (visible light irradiation) effects (C/C_0_) of RhB aqueous solution (10 mg/L) at (**a**) pH = 7 by different catalysts and (**c**) pH = 3–11 by D-BM. The plots of *ln*(C_0_/C) *versus* irradiation time of RhB aqueous solution at (**b**) pH = 7 by different catalysts and (**d**) pH = 3–11 by D-BM. The corresponding adsorption spectra (**e**) and photodegradation ((**f**), cycled catalyst) of RhB solution after 60 min in dark and 40 min irradiation with D-BM at pH = 7. The adsorption (in dark) and photodegradation (visible light irradiation) effects (C/C_0_) of MB aqueous solution (10 mg/L) at (**g**) pH = 3–11 by D-BM and (**h**) the corresponding decomposition rate by different catalysts. (**i**) The absorption spectra of the catalysts. (**j**) Relevant kinetics mechanism of adsorption and photocatalysis for D-BM in RhB solution. (**k**) Schematic diagram of the charge transfer and separation mechanism for the D-BM heterostructure.

To demonstrate the photocatalytic ability of the D-BM heterostructures, photodegradation of RhB in aqueous solution has been investigated under visible-light irradiation. In Fig. 7a, at pH = 7, the corresponding decomposition rate of bare Bi_2_S_3_ (26%) and MoS_2_ (50%) are indistinctive after 40 min. Whereas, when the MoS_2_ was introduced to Bi_2_S_3_, the decomposition rate significantly increases to near 92%. The time-dependent absorption spectra of RhB solutions by D-BM and others were shown in Fig. 7e and Fig. S6. It could conclude that the hierarchical D-BM structure owns outstanding photocatalytic performance than two others. As it has been proposed by Daage, the “rim-edge” mode of MoS_2_ have massive dege active sites, where strong interaction occurs with dye molecules^51^,^52^,^53^. Also, these ultrathin and wrinkled surfaces of MoS_2_ may lead to several internal intersections, which make it possible to bring much more effective area for absorption. Likewise, it is favoring to transfer excited carries between MoS_2_ and Bi_2_S_3_ heterostructure, leading to reduce the recombination efficiency and prolong the lifetime of carries. In addition, the photocatalytic activity can also be related to the amount of the coated MoS_2_ nanosheets, which highlights the optimization of 5 MBS. As for 2 MBS samples, fewer MoS_2_ nanosheets cannot provide enough multiplex refraction for the incident path, as well as the more active site for the adsorption of reactant molecules. On the other hand, 8 MBS samples with superabundant MoS_2_ nanosheets impeded the transformation of photogenerated carries in reverse, thus facilitating their recombination. To make a more specific comparison, the reaction kinetics of all catalysts have been linear fitted as ln (*C*_0_/*C*) = *kt* according to Landmuir-Hinshelwood mechanism shown in Fig. 7b 54,55,56. Note that C and C_0_ are the real-time concentrations and initial concentration of RhB, *t* and *k* denote the irradiation time and the overall photodegradation rate constant, respectively. The rate constant of pristine Bi_2_S_3_ and MoS_2_ are 0.007 and 0.016 min^−1^. Nevertheless, a dramatic improvement (0.073 min^−1^) of 5 MBS can be achieved. Specifically, the increased reaction rates were attributed to the unique porous structure, with faster mass transport and more accessible active sites, resulting in an increased reaction rate. The stability and reusability of the composites (sample 5 MBS at pH = 7) also have been evaluated under irradiation by collecting and reusing them over 3 cycles. As shown in Fig. 7f, the insignificant decline in photocatalytic activity after three runs (90%) confirms the stability of the catalysts, excepting the incomplete collection. The constancy can be recognized through the XRD result after photocatalytic tests (Fig. S7).

It is believed that the effective photocatalysis need handle both the mass transfer and the light transfer issues. The adsorption capacity of catalyst surface for dyes during illumination is a defining factor in photodegradation. In addition, the initial pH plays a dominant role to the adsorption process. Therefore, based on the above adsorption behavior of the D-BM product in dark and neutral surroundings, the pH-dependent adsorption (3.0–11.0) and photocatalysis survey have been conducted. Note that the initial pH of the RhB solution was adjusted by HCl and NaOH solution (1 M). The Fig. 7c reveals that the adsorption capacity of D-BM hybrids has been promoted under acidic conditions and pH = 5 appears to the most beneficial. The adsorption efficiency reaches approximately 76% (Fig. S8) and photodegradation rate *k* = 0.157 min^−1^ (Fig. 7d) at pH = 5. For verifying the adsorption behavior and photocatalytic activity of D-BM hybrid, the colors of the degraded MB solutions (Fig. S8) and photodegradation of MB solution under pH of 3, 5, 7, 9, and 11 have been obtained in Fig. 7g,h. Obviously, D-BM shows a superior adsorption and photodegradation properties compared with pure Bi_2_S_3_ and MoS_2_, especially when pH < 7. Accordingly, although the initial pH affects the adsorption process of dyes onto the catalyst, the photodegradation process of D-BM remains resultful at a large range of pH values. It indicates that D-BM hybrid can serve as a high efficiency catalyst for wastewater treatment, which contained a mild acid commonly.

**Figure 8:**
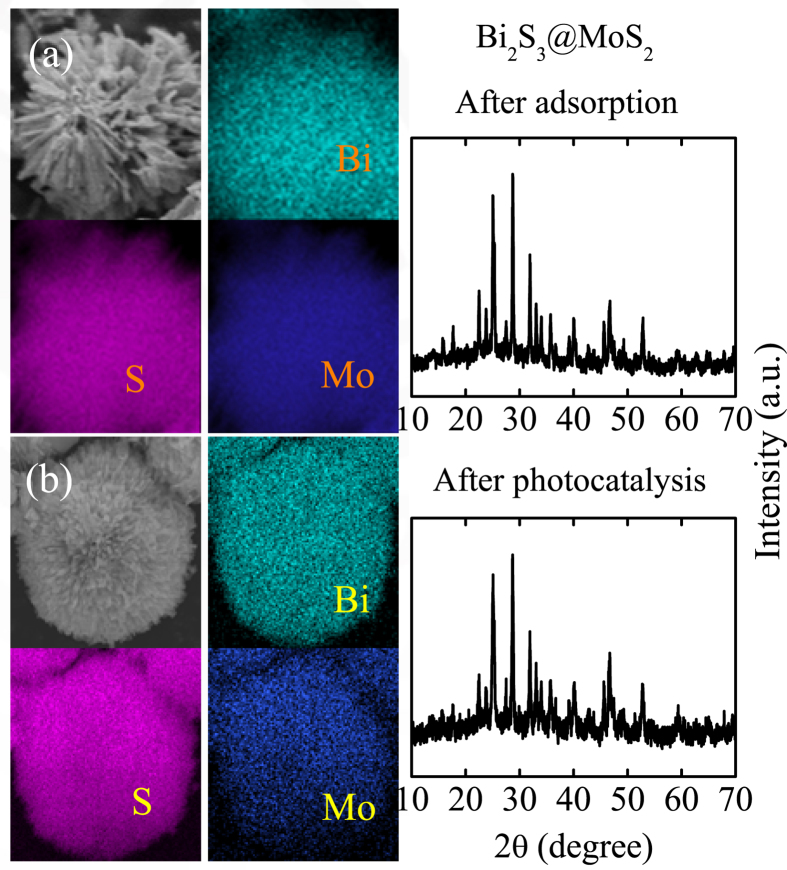
The EDS mapping and the corresponding XRD results after (**a**) adsorption and (**b**) photodegradation tests.

In order to further investigate the stability of the catalyst, the XRD and EDS mapping results after adsorption and photodegradation tests have been shown in Fig. 8a,b. Compared with the XRD results (Fig. 1a) and EDS mapping (Fig. 3e) before photocatalysis, there exist minor change among the elemental composition, spatial distribution, and the phase characterization results. Therefore, the composite has presented relative stability in view of its unique heterostructure and synergistic effect attributed to the intense interaction between Bi_2_S_3_ and MoS_2_, which favors the separation of the photoinduced carriers. However, a small percentage of the samples might had undergone hydrolysis which was unavoidable, taking the slight decrease through cycling experiments and photocorrosion into consideration.

### Relevant kinetics mechanism

In Fig. 7j,k, the related adsorption and photocatalysis kinetic mechanism of the D-BM heterostructure under visible light irradiation has been put forward. In mild acidic environment (pH < 7), the surfaces of the catalyst is exposed to acidic (positive) conditions and positively charged, as shown in Fig. 7j. In addition, both the *π*-bond of RhB and MB can beneficially excite electrons with irradiation, as well as the existence of the functional groups (-COO^−^) as the shown molecular structure of RhB in Fig. 7j. Thus, we can conclude that the dyes might be adsorbed by D-BM hybrid through complex relationship involving electrostatic attraction or hydrogen bonds. Subsequently, these adsorbed dyes on the surface of D-BM could be *in situ* degraded promptly (Fig. 7j). The mass transfer and the chemical reaction process can be guaranteed by the opposite concentration difference and the visible light irradiation, which help to accomplish the degradation synergistically. In order to make it clear, the right part of the Fig. 7j illustrates the complete process simply, which contains both the adsorption and the photocatalysis behaviors of the D-BM hybrid.

On the other hand, since the band-edge potential levels play a crucial role in determining the migration directions of the photoexcited carriers in a heterojunction, the relative band positions of the two semiconductors have been investigated to approach the mechanism of the enhanced photocatalytic activity of their composites. Significantly, a staggered type-II configuration formed at the interface of D-BM structures by the calculated band edge positions, according to the empirical formula: *E*_VB_ = *X* − *E*_e_ + 0.5*E*_g_^12^. Note that *E*_VB_ is the valence band (VB) edge potential and *E*_*e*_ refers to the free electrons energy based on the hydrogen scale (4.5 eV). Based on previous studies, *X* is the electronegativity of the corresponding semiconductor (5.28 eV for Bi_2_S_3_ and 5.32 eV for MoS_2_), expressed as the geometric mean of the electronegativity for the component atoms, and the band gap energy *E*_g_ of Bi_2_S_3_ and MoS_2_ are 1.33 eV and 1.3 eV, respectively^12^,^19^. Correspondingly, the *E*_VB_ and *E*_CB_ (CB, the conduct band) of Bi_2_S_3_ are estimated to be 0.12 eV and 1.45 eV, both higher than those of MoS_2_, with Δ*E*_VB_ = 0.02 eV and Δ*E*_CB_ = 0.05 eV, respectively. Compared with the pure samples, the heterostructure of D-BM presents an obvious enhanced absorption (Fig. 7i), both in range and intensity. As illustrated in Fig. 7k, under visible-light irradiation, excited electrons-holes are generated facilely in both Bi_2_S_3_ and MoS_2_. With the suitable type II band alignment, the transfer of carries are also performed from the CB of Bi_2_S_3_ to MoS_2_, as well as the reflux of holes mainly from the VB of MoS_2_ to Bi_2_S_3_. Therefore, more vacancies have been left at the bottom of CB and top of VB for Bi_2_S_3_/MoS_2_, resulting in pronging the lifetime of carries and boosting more available separation. After the photoexcited carries fleetly flow to the CB of MoS_2_, the reaction of O_2_ → O_2_•^−^ has been further promoted through one-electron reducing. Then hydroxyl radicals OH•, the dominating active species of RhB photodegradation, can be generated by the reaction between water and the unstable superoxide radical anion O_2_•^− ^57. Meanwhile, the remaining holes in the valence band of Bi_2_S_3_ also participate in the degradation of RhB as the trapped active species.

### Photoconductance and photoresponse performances

**Figure 9:**
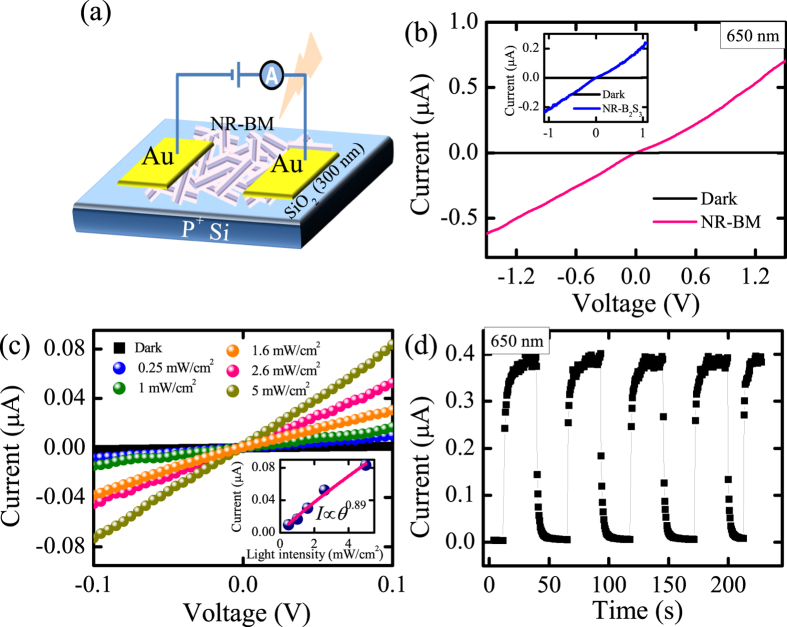
(**a**) Schematic illustration of the fabricated photodetector. The I-V curves of the NR-BM (Inset of Fig. 8b for NR-Bi_2_S_3_) photodetectors illuminated by 650 nm with (**b**) 1.0 mW/cm^2^ and (**c**) different incident light intensity. Inset of Fig. 8c is the photocurrent measured *versus* light density at a bias voltage of 0.1 V. (**d**) The time dependent of the on-off photocurrent response of the device at a bias of 1 V by 650 nm illumination.

Besides photocatalysis, the as-synthesized D-BM hybrid is expected to have improved photoconductance and photoresponse properties compared with the pure Bi_2_S_3_ structure. In order to spin coating, the photodetector devices (Fig. 9a) were fabricated by these disrupt NR-BM and Bi_2_S_3_ nanorods (NR-Bi_2_S_3_). Figure 9b plots the typical current-voltage (*I*–*V*) curves of NR-BM based nano-photodetector exposed to 650 nm illumination (1.0 mW cm^−1^) and in dark, respectively. It is obvious that the quasilinearity of the *I*–*V* curves indicates the formation of an good Ohmic contact between Au electrode and NR-BM interface. With illumination, the NR-BM shows a remarkable photocurrent reaches about 0.703 *μ*A under −1.5 V bias voltage, resulting a higher *I*_light_/*I*_dark_ ratio to about 567, which compared to the NR-Bi_2_S_3_ of 126 with the shortened lifetime of the electron-hole pairs (Inset of Fig. 9b). The electrical characteristics of the NR-BM based device have been investigated in dark and at increasing illumination intensities from 0.25 mW cm^−1^ to 5 mW cm^−1^ (Fig. 9c). Clearly, the photocurrent increases with the enlarged light intensity and exhibits a high dependence on it, and their relationship can be described by a simple power law as I = *AP*^θ ^58,59. Where *A* and θ represent a constant for a certain wavelength and the response of photocurrent to light intensity, respectively. The fitting curve leads to θ = 0.89, which suggests that there exists little trap states (Inset of Fig. 9c) in the NR-BM photodetector. In addition, Fig. 9d provides the time dependent photoresponse of the D-BM device at a bias voltage of 1 V, which sheds light on a readily switch between high- and low-conduction states by illumination on/off. It is known that the responsivity (*R*) serves as a critical metric to the photodetector sensitivity, which is defined as R(AW^−1^) = *I*_p_/*P*_opt_^60^. Where *I*_p_ and *P*_opt_ are the photocurrent and incident light power, respectively. Based on the results, the *R* of the D-BM device is estimated to be 13.3 AW^−1^. Consequently, the improvement of photoconductivity and photoresponse performance supports the efficient carries separation resulting from the heterostructures of dandelion-shaped Bi_2_S_3_@MoS_2_.

### Theoretical investigation

**Figure 10:**
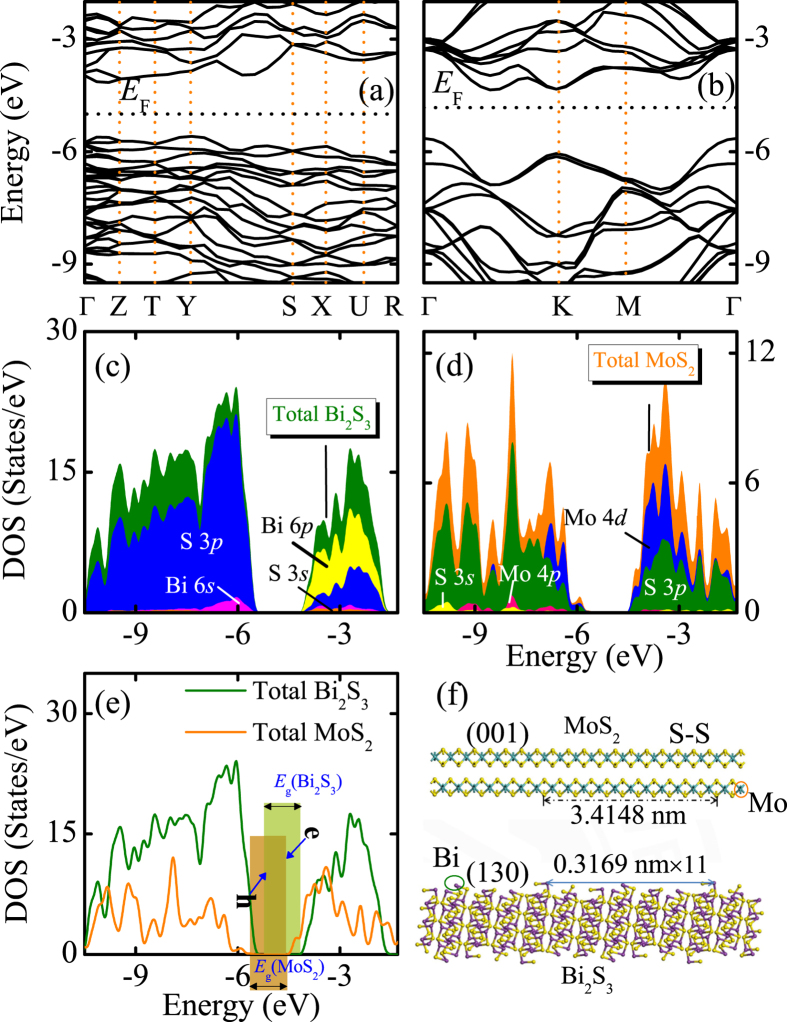
Calculated band structures (**a**,**b**), the density of states (**c**–**e**), and the crystal surface matching between the (130) and (001) crystal surfaces (**f**) of Bi_2_S_3_ and MoS_2_, respectively.

The obtained band structures and the density of states (DOS) of Bi_2_S_3_ and MoS_2_ have been shown in Fig. 10a–f, respectively. As we can see, the top of VB contains S 3*p* and few contributions of Bi 6*s*, while the bottom of CB are mostly Bi 6*p* and S 3*p* for Bi_2_S_3_, with calculated *E*_g_ = 1.44 eV. As for MoS_2_ (with calculated *E*_g_ = 1.29 eV), the top of VB originates from Mo 4*d*, and the bottom of CB are Mo 4*d* and some hybridization with S 3*p*. Note that Fig. 10f shows the crystal surface matching of MoS_2_ (top) and Bi_2_S_3_ (down). By aligning the Fermi level relative to the vacuum energy level, the obtained work functions for Bi_2_S_3_ and MoS_2_ are 4.88 and 5.00 eV, respectively. Due to the differential work functions, a built-in electric field from Bi_2_S_3_ to MoS_2_ can be established near the interface. Thus the built-in electric field of the composites is expected to facilitate the separation of photo-generated carriers. It can be concluded that the theoretical calculations of the band energy positions (Fig. 10e) keep highly unanimous with the experimental results. In consequence, the MoS_2_ nanosheets uniformly layered-coated the Bi_2_S_3_ microspheres sufficiently absorb visible-light and retard the electron-hole recombination, eventually leading to improvement of the photocatalytic and optoelectronic properties. Moreover, the unique novel architecture can provide valuable references to take advantage of solar energy in the future.

## Conclusion

In summary, a green strategy based on the hydrothermal method has been developed for the fabrication of hierarchical 3D dandelion-shaped Bi_2_S_3_ microspheres coated with layered MoS_2_ nanosheets. The novel heterostructure with unique porosity and intimate interfacial contact can provided efficient visible-light utilization and penetrable paths for reactant molecules to reach the inner structure. Compared with the pristine Bi_2_S_3_ or MoS_2_, the as-synthesized D-Bi_2_S_3_@MoS_2_ composite has exhibited much higher adsorption behavior and photocatalytic activity under visible-light irradiation. The formed staggered type II band alignment of Bi_2_S_3_@MoS_2_ has expected to promote the separation of carries, accelerate the transportation and prolong lifespan of electron-hole pairs, which can be verified by the excellent photoconductance and photoresponse properties. Correspondingly, the Bi_2_S_3_@MoS_2_-0.5 mol (5 MBS) has been proven to achieve an optimal photocatalysis performance, highlighting the importance of novel core/shell heterostructures for environmental remediation and solar energy harvesting applications in the future.

## Methods

### Synthesis

Generally, the D-BM heterostructure were synthesized by a two-step hydrothermal method. Firstly, the dandelion-shaped Bi_2_S_3_ microspheres were prepared at 180 °C^19^. Then, for forming the hybrids, another hydrothermal process was employed to embed MoS_2_ nanosheets onto Bi_2_S_3_ microspheres (molar ratios of Mo^4+^ to Bi^3+^ were 20%, 50%, and 80%, with the labels of 2 MBS, 5 MBS, and 8 MBS, respectively). Further information about the experimental details are available in the ESI.

### Characterization

The crystallinity and purity of the resulting products were assessed by X-ray diffraction (XRD, Bruker D8 Advance diffractometer) equipped with Cu Kα radiation (*λ* = 1.5418 *Å*). Field emission scanning electron microscopy (FESEM, JEOL-JSM-6700F) was employed to investigate the morphologies of the synthesized samples, equipped with an energy dispersive X-ray spectroscopy (EDS). For detailed insight into the 2D-layered MoS_2_ coated Bi_2_S_3_, Transmission Electron Microscope (TEM) and high resolution transmission electron microscopy (HRTEM) studies were analyzed at the accelerating voltage of 200 kV. X-ray photoelectron spectroscopy (XPS, RBD upgraded PHI-5000C ESCA system, Perkin-Elmer) measurements were carried out with Mg-Kα radiation (*h*ν = 1253.6 eV). Raman spectroscopy experiments were implemented by Jobin-Yvon LabRAM HR 800 micro-Raman spectrometer using a 532 nm line from a He-Cd laser. The absorption spectra have been obtained using the PerkinElmer Lambda 950 spectrophotometer in dilute solution. The specific surface area were calculated by the Brunauer-Emmett-Teller (BET) method (TriStar II 3020, America).

### Photocatalytic test

The photocatalytic performance of the as-synthesized D-BM composites were evaluated by degrading RhB and MB (organic pollutant of dye wastewater) under visible light irradiation. More details about the photocatalytic tests have been provided in ESI. In addition, transient photocurrent responses for the as-prepared catalysts were performed over an electrochemical analyzer (CHI660D Instruments, China) in a standard three electrode system. Under the irradiation of simulated sunlight (500 W Xe lamp with a cutoff filter), the prepared samples acted as working electrode (ITO as supporter). A Pt wire worked as counter electrode, and Ag/AgCl (saturated KCl) as reference.

### Measurement of photoconductance

The photoconductance and photoresponse behavior of the D-BM was studied based on the nano-photodetectors. Typically, the disrupt Bi_2_S_3_@MoS_2_ nanorods (NR-BM) from D-BM microspheres by oscillating (Sonics VCX800, America) were drop-deposited on the SiO_2_ (300 nm)/Si substrate. Then the Au electrodes (100 *μ*m × 150 *μ*m) were defined on the NR-BM film by photolithography and high-vacuum electron beam (EB) evaporation process. The optoelectronic properties of the fabricated device were measured by a semiconductor parameter analyzer system (Keithley 4200-SCS) with the illumination laser of 650 nm.

### Theoretical calculation

In the present work, the first-principles calculations of the heterogeneous structure have been performed, to provide the theoretical basis of the promoted photocatalytic activity. In details, the plane-wave pseudopotential calculations with the generalized gradient approximation (GGA) of Perdew-Burke-Ernzerhof (PBE) have been carried out using the Materials Studio 7.0 Package^61^,^62^,^63^. The cutoff kinetic-energy (Bi_2_S_3_ and MoS_2_) are 600 eV/450 eV and the 3 × 8 × 3/5 × 5 × 1 Monkhorst-Pack *k*-point mesh have been employed for the Brillouin-zone integration. The electronic structures were calculated with the optimized lattice geometries. Based on the convergence criteria, both energy and force were less than 10^−5^ eV and 0.01 eV Å^−1^, for the fully relaxed initiating structures, respectively.

## Additional Information

**How to cite this article**: Li, M. *et al*. Superior adsorption and photoinduced carries transfer behaviors of dandelion-shaped Bi_2_S_3_@MoS_2_: experiments and theory. *Sci. Rep.* **7**, 42484; doi: 10.1038/srep42484 (2017).

**Publisher's note:** Springer Nature remains neutral with regard to jurisdictional claims in published maps and institutional affiliations.

## Supplementary Material

Supporting Information
