# Supplementary material for: Superior adsorption and photoinduced carries transfer behaviors of dandelion-shaped Bi2S3@MoS2: experiments and theory
Source: Sci Rep. 2017 Feb 13;7:42484. doi: 10.1038/srep42484 (PMC5304175; doi:10.1038/srep42484)
Supplement: Supporting Information [file srep42484-s1.pdf]

Electronic Supplementary Information

Superior adsorption and photoinduced carries transfer behaviors of dandelion-shaped Bi_2S_3@MoS_2: experiments and theory

Mengjiao Li, Junyong Wang, Peng Zhang, Qinglin Deng, Jinzhong Zhang, Kai Jiang, Zhigao Hu[a], and Junhao Chu

Technical Center for Multifunctional Magneto-Optical Spectroscopy (ECNU), Shanghai & Department of Electronic Engineering, East China Normal University, Shanghai 200241, China

[a]Author to whom correspondence should be addressed.

Tel.: +86-21-54345150. Fax: +86-21-54345119.

Electronic mail: zghu@ee.ecnu.edu.cn

Further details for preparation and photocatalytic activity test of the catalysts.

Synthesis of D-Bi_2S_3 microspheres Three-dimensional dandelion-shaped Bi_2S_3 microspheres were prepared by a facile hydrothermal method. In the typical process, a certain amount of polyethylene glycol (PEG-4000) was dissolved in 30 ml of deionized water (DI) and stirred uniformly. Then $Bi(NO_3)_3 \cdot 5H_2O$ (3 mmol) and thiourea (10 mmol) were successively added into the above solution, with constant stir. Finally, the homogeneous yellowish solvent was transferred into a 50 mL Teflon-lined stainless steel autoclave and hydrothermally treated at 180 °C. After 10 h, the Teflon vessel was cooled down to room temperature and the black product was collected. Then urchin-shaped Bi_2S_3 microspheres were obtained after being washed with DI and anhydrous ethanol several time and dried overnight at 60 °C in a vacuum oven.

Fabrication of D-Bi_2S_3@MoS_2 (D-BM) microspheres To synthesize the D-BM heterostructure, another hydrothermal process was employed. Firstly, a certain quality of as-synthesized D-Bi_2S_3 was dispersed into 40 ml of DI under ultrasonication to form a suspension A, followed by adding quantitative oxalic acid (for PH<7) into it. Then sodium molybdate ($Na_2MoO_4 \cdot 2H_2O$) (1 mmol) and thiourea (5 mmol) were dissolved in 20 mL deionized water and stirred well for 30 min as solvent B. Under violent stirring, the solvent B was slowly inject into suspension A for 30 min. Finally, the uniform mixture was transferred into a 100 mL Teflon-lined stainless steel autoclave and heated to 200 °C for 24 h to form the @MoS_2 coated Bi_2S_3 core-shell structure. The molar ratios of Mo^{4+} to Bi^{3+} were 20%, 50%, and 80%, thus the resulting samples were labeled as 2MBS, 5MBS, and 8MBS, respectively. In the same way, the pristine MoS_2 nanoflowers were synthesized excepting for without Bi_2S_3 microspheres.

Photocatalytic Test The photocatalytic performance of the as-synthesized D-BM composites were evaluated by degrading RhB (organic pollutant of dye wastewater) under visible light irradiation. A 500 W Xe lamp with a 400 nm cutoff filter was used as the light sources and it was surrounded by a cooling water cycle system to keep the temperature stable. Typically, 20 mg samples were added to 50 ml of MB aqueous solution (10 mg/L) and the mixed suspension was

kept in the dark under stirring for 60 min to reach an adsorption-desorption equilibrium between the MB and the photocatalysts. Then after exposing to light irradiation, adequate aliquots (3 ml) of the suspension were extracted and centrifuged at 10600 rpm. The degradation of the dye was monitored by checking the absorbance at 554 nm using a UV-vis spectrophotometer (PerkinElmer Lambda 950). Transient photocurrent responses for the as-prepared catalysts were performed over an electrochemical analyzer (CHI660D Instruments, China) in a standard three electrode system. Under the irradiation of simulated sunlight (500 W Xe lamp with a cutoff filter), the prepared samples acted as working electrode (ITO as supporter). A Pt wire worked as counter electrode, and Ag/AgCl (saturated KCl) as reference.

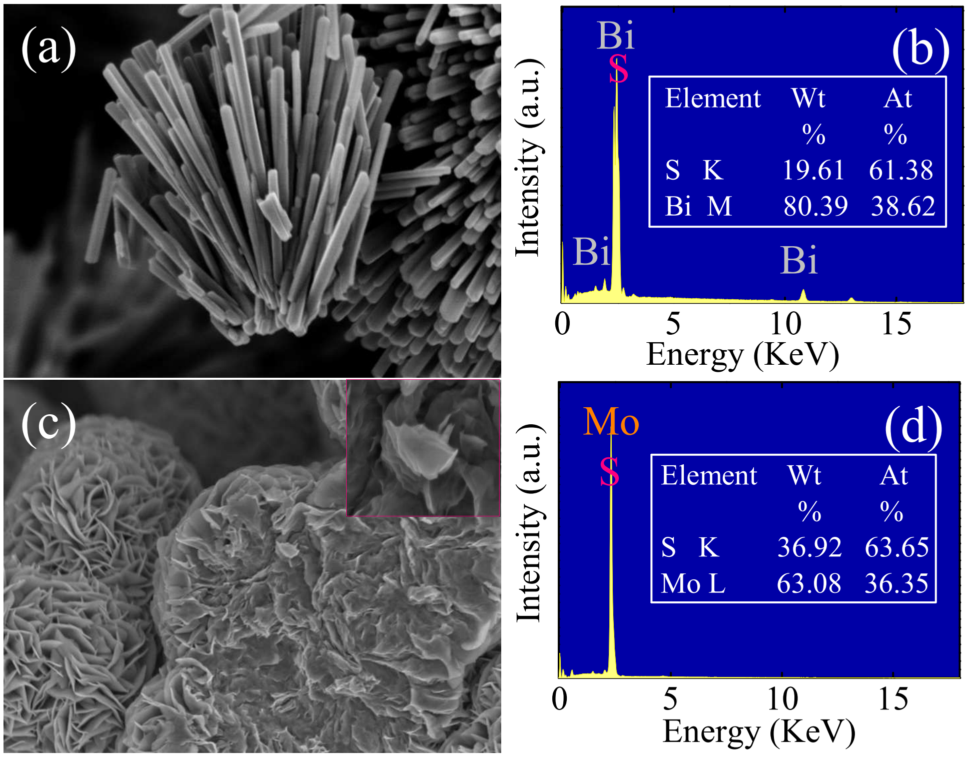

Fig. S 1: (a) The SEM image of a bunch of Bi_2S_3 nanorods (c) The SEM image of the chapped MoS_2 flowers and the inset is MoS_2 nanosheets. EDS spectra of (b) D-Bi_2S_3 microspheres and (d) MoS_2 microflowers.

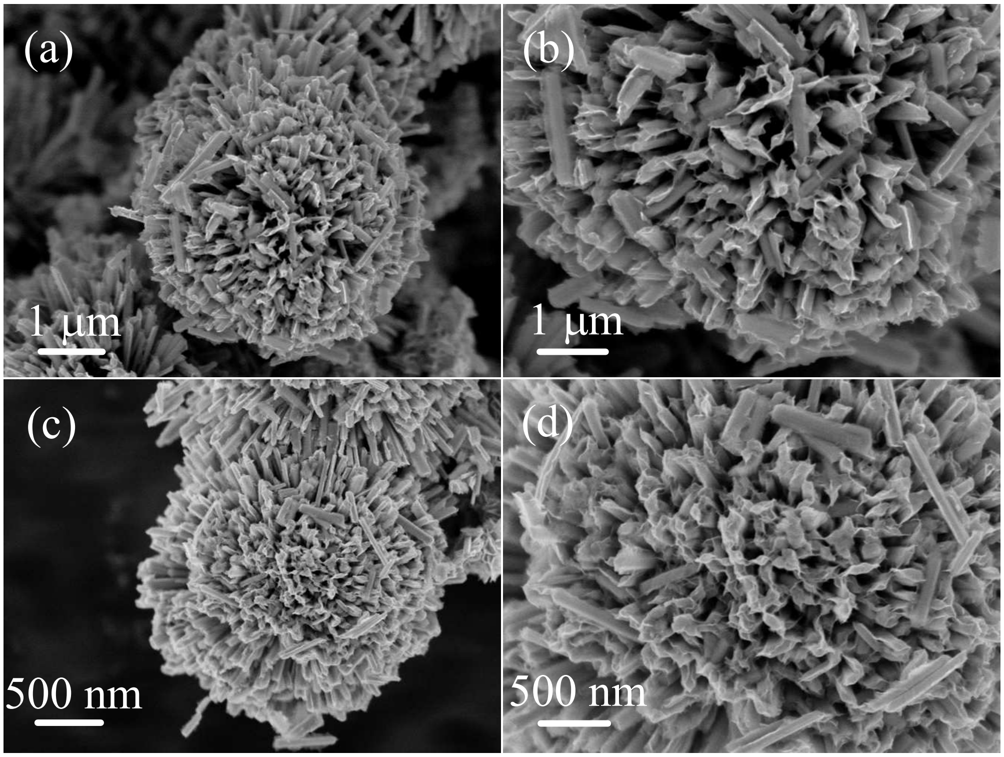

Fig. S 2: Different magnifications of the D-BM hybrids for (a)-(b) 2MBS and (c)-(d) 8MBS.

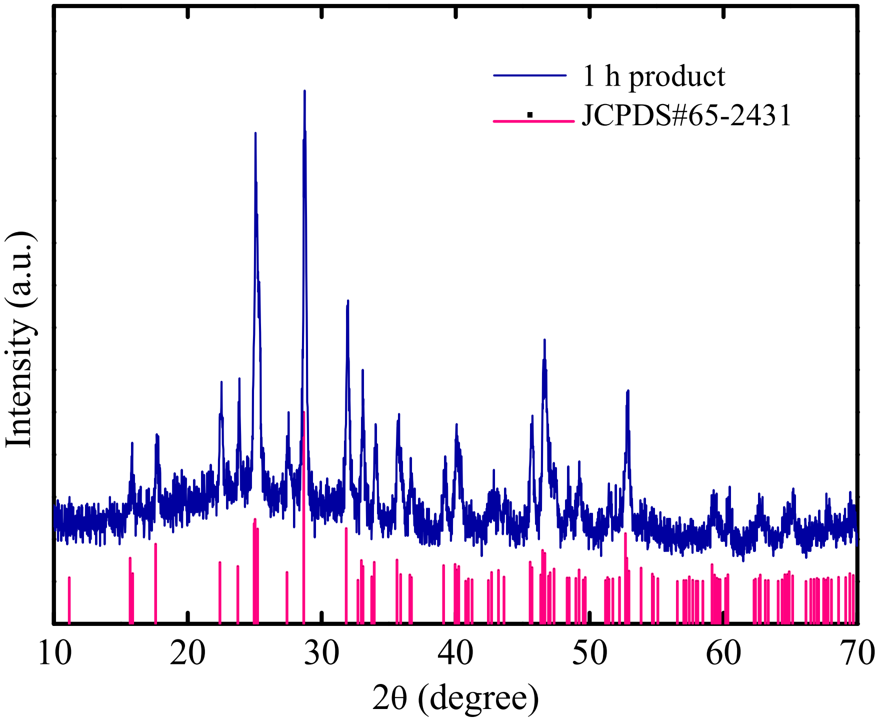

Fig. S 3: XRD pattern of the product at the early reaction stage (1 h) and the corresponding standard pattern for Bi_2S_3 (JCPDS#65-2431).

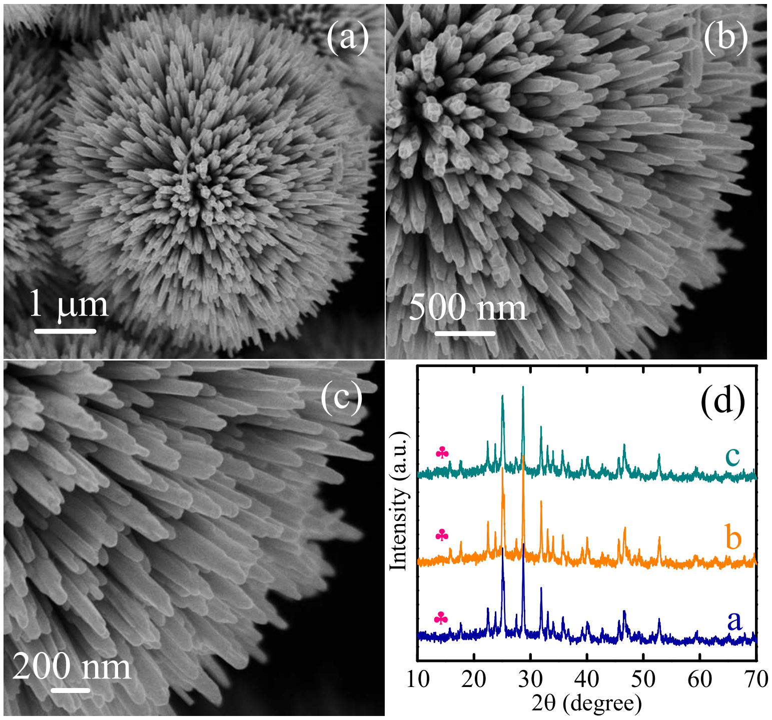

Fig. S 4: (a)-(c) The SEM images of the obtained Bi_2S_3 samples at 3 h with the rough surfaces. (d) The XRD patterns of the products at a-5 h, b-8 h, and c-12 h, respectively.

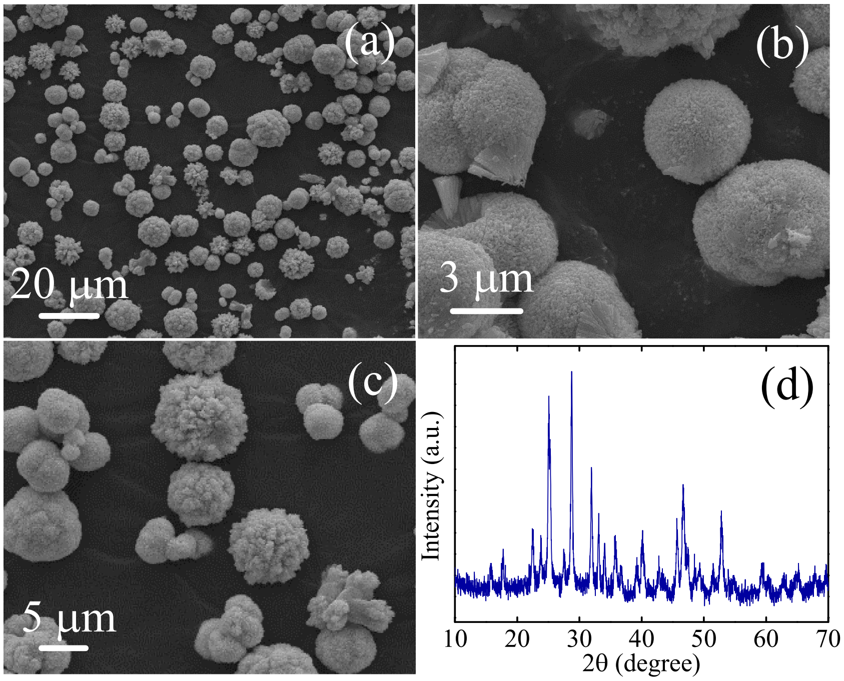

Fig. S 5: (a)-(c) The SEM images of the obtained Bi_2S_3 samples by hydrothermal process without PEG. (d) The XRD patterns of the Bi_2S_3 samples with PEG.

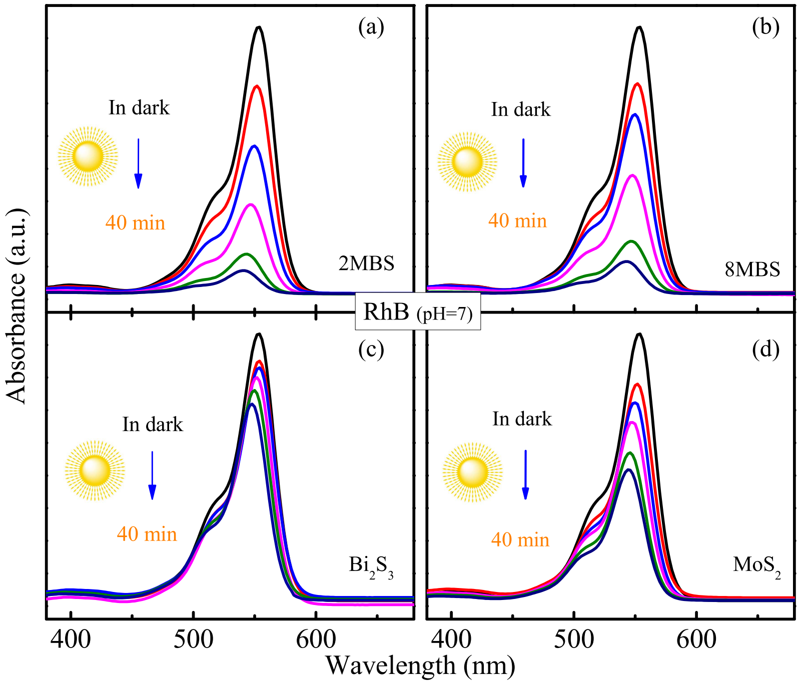

Fig. S 6: (a)-(d) The corresponding adsorption spectra of RhB solution after 60 min in dark and 40 min irradiation at pH=7 with catalysts of 2BMS, 8BMS, Bi_2S_3, and MoS_2, respectively.

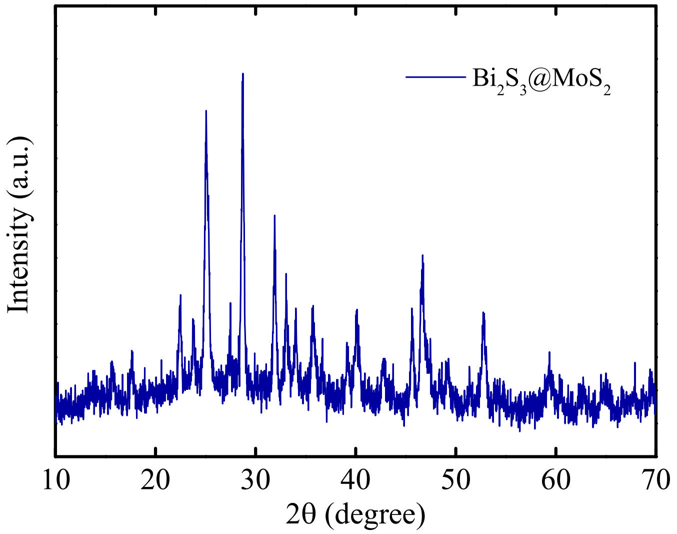

Fig. S 7: The XRD patterns of the D-BM hybrids collected after the photocatalytic degradation for 3 cycles.

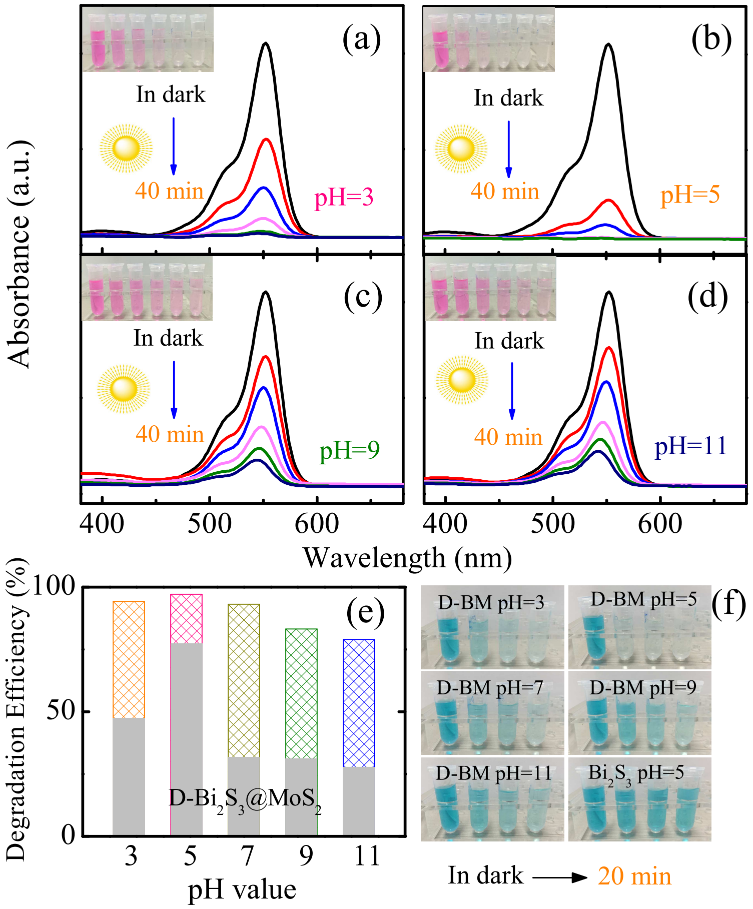

Fig. S 8: (a)-(d) The corresponding adsorption spectra of RhB solution after 60 min in dark and 40 min irradiation with D-BM at pH=3, 5, 9, and 11. (e) The corresponding decomposition rate at different pH values with D-BM. (f) The relevant color of the degraded MB solutions at different pH values.
